# World Pneumonia Day — November 12, 2014

**Published:** 2014-11-07

The sixth annual World Pneumonia Day is being observed November 12, 2014, to raise awareness about pneumonia’s toll and to promote interventions to protect against, treat, and prevent the disease globally. The United States has made great strides in protecting children from the serious, and sometimes deadly, effects of pneumonia through recent vaccination efforts. Tennessee, for example, is experiencing historically low rates of pneumonia hospitalizations in children aged <2 years since pneumococcal conjugate vaccines were introduced in 2000 (1). Data suggest that this progress also is being seen across the country (2). In spite of this success, however, pneumonia still kills approximately 50,000 people in the United States each year, 85% of whom are adults aged ≥65 years. In response, this year CDC recommended pneumococcal conjugate vaccine for adults aged ≥65 years.

Globally, pneumonia kills nearly 1 million children aged <5 years each year (3). In addition to bacterial pathogens, many viruses such as respiratory syncytial virus, influenza, and measles also are major causes of pneumonia globally. Many deaths and illnesses from pneumonia can be prevented with the use of 1) pneumococcal, *Haemophilus influenzae* type b (Hib), influenza, and measles vaccines; 2) appropriate antimicrobial therapy; and 3) supportive health care, among other strategies.

Communities around the world face a range of respiratory disease threats, including reemerging or newly identified pathogens. In late summer, infection with the uncommon enterovirus EV-D68 led to the hospitalization of hundreds of children in multiple states (4). In and around the Arabian Peninsula, a recently recognized coronavirus (Middle East respiratory syndrome coronavirus) has been fatal in about one third of reported cases (5). Vaccines are not available to provide protection against these or many of the other pathogens that commonly cause pneumonia, including respiratory syncytial virus, human metapneumovirus, and *Mycoplasma pneumoniae*, highlighting the importance of research into vaccine development as well as effective treatment and diagnostics for viral and bacterial pneumonia. Additional information regarding World Pneumonia Day is available at http://worldpneumoniaday.org.

## References

[1] Griffin MR, Mitchel E, Moore MR, Whitney CG, Grijalva CG (2014). Declines in pneumonia hospitalizations of children aged <2 years associated with introduction of 13-valent pneumococcal conjugate vaccine—Tennessee, 1998–2012. MMWR Morb Mortal Wkly Rep.

[2] Simonsen L, Taylor RJ, Schuck-Paim C, Lustig R, Haber M, Klugman KP (2014). Effect of 13-valent pneumococcal conjugate vaccine on admissions to hospital 2 years after its introduction in the USA: a time series analysis. Lancet Respir Med.

[3] Liu L, Oza S, Hogan D (2014). Global, regional, and national causes of child mortality in 2000–13 with projections to inform post-2015 priorities: an updated systematic analysis. Lancet.

[4] Midgley CM, Jackson MA, Selvarangan R (2014). Severe respiratory illness associated with enterovirus D68—Missouri and Illinois, 2014. MMWR Morb Mortal Wkly Rep.

[5] CDC (2013). Updated information on the epidemiology of Middle East respiratory syndrome coronavirus (MERS-CoV) infection and guidance for the public, clinicians, and public health authorities, 2012–2013. MMWR Morb Mortal Wkly Rep.

